# Characterizing Benzo[*a*]pyrene-induced *lacZ* mutation spectrum in transgenic mice using next-generation sequencing

**DOI:** 10.1186/s12864-015-2004-4

**Published:** 2015-10-19

**Authors:** Marc A. Beal, Rémi Gagné, Andrew Williams, Francesco Marchetti, Carole L. Yauk

**Affiliations:** Carleton University, Ottawa, ON K1S 5B6 Canada; Environmental Health Science and Research Bureau, Healthy Environments and Consumer Safety Branch, Health Canada, Ottawa, ON K1A 0K9 Canada

**Keywords:** Mutation spectra, Next-generation sequencing, Transgenic rodent assay, Benzo[a]pyrene, Muta™Mouse

## Abstract

**Background:**

The transgenic rodent mutation reporter assay provides an efficient approach to identify mutagenic agents *in vivo*. A major advantage of this assay is that mutant reporter transgenes can be sequenced to provide information on the mode of action of a mutagen and to identify clonally expanded mutations. However, conventional DNA sequence analysis is laborious and expensive for long transgenes, such as *lacZ* (3096 bp), and is not normally implemented in routine screening.

**Methods:**

We developed a high-throughput next-generation sequencing (NGS) approach to simultaneously sequence large numbers of barcoded mutant lacZ transgenes from different animals. We collected 3872 mutants derived from the bone marrow DNA of six Muta™Mouse males exposed to the well-established mutagen benzo[a]pyrene (BaP) and six solvent-exposed controls. Mutants within animal samples were pooled, barcoded, and then sequenced using NGS.

**Results:**

We identified 1652 mutant sequences from 1006 independent mutations that underwent clonal expansion. This deep sequencing analysis of mutation spectrum demonstrated that BaP causes primarily guanine transversions (e.g. G:C → T:A), which is highly consistent with previous studies employing Sanger sequencing. Furthermore, we identified novel mutational hotspots in the lacZ transgene that were previously uncharacterized by Sanger sequencing. Deep sequencing also allowed for an unprecedented ability to correct for clonal expansion events, improving the sensitivity of the mutation reporter assay by 50 %.

**Conclusion:**

These results demonstrate that the high-throughput nature and reduced costs offered by NGS provide a sensitive and fast approach for elucidating and comparing mutagenic mechanisms of various agents among tissues and enabling improved evaluation of genotoxins.

**Electronic supplementary material:**

The online version of this article (doi:10.1186/s12864-015-2004-4) contains supplementary material, which is available to authorized users.

## Background

Transgenic mutation reporter rodent models, such as Muta™Mouse [1], enable the detection of spontaneous and induced mutations *in vivo* (reviewed in ref. [2]) and the identification of chemical mutagens. These transgenic rodents carry neutral reporter transgenes that can be easily recovered and detected in a bacterial host when mutated. Studies using the transgenic rodent (TGR) mutation reporter assay are very efficient and commonly used. The Organisation for Economic Co-operation and Development (OECD) has recently established guidelines for mutagenicity testing using this assay [3]. One major advantage of the TGR assay is that mutant transgenes can be sequenced to provide mechanistic insight into spontaneous and induced mutagenesis. DNA sequence analysis of mutations offers several major benefits in genetic toxicology: 1) mutation spectrum identifies the target nucleotides and types of mutations providing information on the mutagen’s mode of action; 2) it can be used to correct for clonal expansion of mutant transgenes for a more accurate calculation of mutation frequency (versus mutant frequency); 3) it allows for a full characterization of mutational hotspots across a specific locus; 4) it permits a comparison of mechanisms of mutagenesis operating in different target tissues; and 5) differences in mutation spectra between treated and control groups can further support a mutagenic effect, especially for weak mutagens. Thus, sequence analysis provides the TGR assay with a depth of information that is not available in most genotoxicity assays.

One of the most commonly used reporter transgenes is the bacterial *lacZ* gene because there is a simple, efficient, and robust positive selection method for identifying *lacZ* mutations in *Escherichia coli (E. coli)* [4]. Mutant detection is conducted by extracting DNA from the tissues of exposed *lacZ*-carrying transgenic rodents, packaging of the *lacZ* transgene into bacteriophage and infecting host *E. coli*. Under selective conditions, only phage particles with mutated *lacZ* genes will be able to form plaques. Mutant frequency is calculated as the proportion of mutant plaques versus total plaques under non-selective conditions. The *lacZ* coding sequence is the longest of the mutation reporter transgenes (3096 bp) and thus has many target nucleotide positions where mutations can induce the mutant phenotype, making it a very sensitive reporter. However, the length of the *lacZ* gene poses challenges for sequence analysis of mutants. For this reason, few studies have sequenced the *lacZ* transgene to characterize mutation spectra following mutagen challenge [4–10]. Sequencing *lacZ* requires a complementation assay to narrow down the mutation location, followed by several Sanger sequencing reactions of the section containing the mutant [5, 6, 9, 10]. Therefore, the number of mutants that can be sequenced in an experiment is limited and most studies generally only sequence a few hundred mutants in total across their experimental groups. The majority of studies conducting mutation spectrum analysis use different and much shorter mutation reporter genes (*cII,* 294 bp; *gpt*, 456 bp; *lacI*, 1080 bp) because they are small enough to be sequenced using Sanger sequencing.

Next-generation sequencing (NGS) had been used to target and sequence pools of *cII* mutants from Big Blue® mice [11]. These authors pooled 150 *cII* mutants per sample and then sequenced 1776 mutations using 454 pyrosequencing to characterize the mutation spectra of ultraviolet radiation, 4-aminobiphenyl, and second-hand smoke, demonstrating the power of using NGS to sequence reporter genes. However, the short *cII* transgene is not as sensitive to induced mutations as longer genes and is therefore used much less frequently for routine screening (only ~2 % of studies using Big Blue® or Muta™Mouse actually measure mutations in the *cII* gene [2]). With the advent of NGS, targeted sequencing of large fragments is now possible [12], and with the high-throughput nature of this technology, thousands of long fragments, such as the *lacZ* gene, can be sequenced in a single run. Thus, NGS can be adapted to identify mutations within longer reporter genes enabling the routine incorporation of mutation spectrum analysis into the standard TGR assay.

Here we developed a high-throughput and cost-effective approach that uses NGS to characterize *lacZ* mutation spectra in the Muta™Mouse model. We collected *lacZ* mutants from the bone marrow of control and benzo[a]pyrene (BaP) treated mice. BaP is a model mutagen known to form bulky DNA adducts with guanine bases, inducing primarily G:C **→** T:A transversions [6, 13–17]. Barcoding these individual mutant plaques would allow for the sequence from each plaque to be followed independently. However, this approach would not be any more efficient or cost-effective than Sanger sequencing individual mutants (Sanger: PCR amplification of multiple amplicons for sequencing vs. NGS: building a library). In the present work, we pooled mutants from each animal in a mixture, barcoded the mixture, and sequenced all the mutants in that mixture at the same time under the same barcode using an Ion Proton™ sequencer. This approach is much more efficient but does not allow us to track the mutations within a single plaque. Rather than assigning mutants to a specific plaque, mutations are identified within the pool and considered together as representative of the mouse sample. Using this approach, we enriched and sequenced an unprecedented 3872 *lacZ* mutant plaques from control and BaP-exposed samples in a single sequencing run and established the BaP-induced mutation spectrum with unparalleled precision. We developed an analytical pipeline to reduce false positive mutants (PCR and sequencing artefacts) that includes the use of technical replicates and stringent filtering approaches. We applied our pipeline to determine the degree of clonal expansions and make adjustments to determine mutation frequency within our population of mutants for each mouse, to precisely characterize spontaneous and BaP-induced mutation spectrum, and to determine the mutational hotspots across the *lacZ* locus in both control and BaP-exposed mice.

## Methods

### Animal treatment

Six Muta™Mouse males (approximately 15 weeks of age; strain 40.6 carrying 29.0 ± 4.0 copies of *lacZ* on chromosome 3 [18]) were exposed daily to BaP dissolved in olive oil by oral gavage for 28 days (100 mg/kg body weight/day). An additional six males received olive oil and served as the vehicle controls. Three days after the last treatment, mice were anaesthetized with isofluorane and euthanized via cervical dislocation. Bone marrow was isolated from the mice by flushing the femurs with 1X phosphate-buffered saline. After brief centrifugation and supernatant removal, the pellet was flash-frozen in liquid nitrogen and then stored at −80 °C. All animal procedures were carried out under conditions approved by the Health Canada Ottawa Animal Care Committee.

### Muta™Mouse *lacZ* mutation analysis

Bone marrow was thawed, digested in 5 mL of lysis buffer (10 mM Tris–HCl, pH 7.6, 10 mM ethylenediaminetetraacetic acid (EDTA), 100 mM NaCl, 1 % sodium dodecyl sulfate (*w/v*), 1 mg/mL Proteinase K), and incubated at 37 °C overnight with gentle shaking. Genomic DNA was then isolated using a phenol/chloroform extraction procedure described previously [4, 5]. DNA was dissolved in 100 μL of TE buffer (10 mM Tris pH 7.6, 1 mM EDTA) and stored at 4 °C for several days before use. *LacZ* transgene mutants present in the genomic DNA were identified using the Phenyl-β-D-galactopyranoside (P-gal) positive selection assay [4]. Briefly, the λgt10*lacZ* construct was packaged into phage particles through the cohesive ends (cos sites) using the Transpack™ lambda packaging system (Agilent, Santa Clara, CA). Packaged phage particles were subsequently mixed with the host bacterium (*E. coli lacZ*^−^, *galE*^−^, *recA*^−^, pAA119 with *galT* and *galK*) [19] in order to infect the *E. coli* with the *lacZ* construct. The *E. coli* were then plated on minimal medium containing 0.3 % (*w/v*) P-gal and incubated overnight at 37 °C. P-gal is toxic to *galE*^−^ strains that express a functional copy of *lacZ*, and thus, only *E. coli* receiving a mutated copy of the *lacZ* transcript will form plaques on the P-Gal medium. Packaged phage particles were concurrently plated on titre plates without P-Gal to determine the total plaque-forming units (pfu). The *lacZ* mutant frequencies were then calculated by determining the ratio of mutant pfu to total pfu. The experimental design and *lacZ* mutation analysis used in this study followed OECD guidelines [3]. For more information on the *lacZ* mutation assay, there is a recently published video describing the assay and procedural steps used here [20].

### *lacZ* mutant plaque collection and preparation for next-generation sequencing

**Fig. 1:**
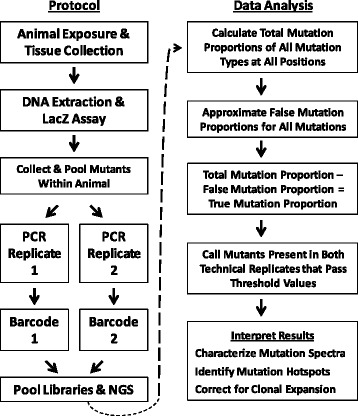
Brief overview of workflow used in this study

The procedure used by Besaratinia et al. [11] to amplify *cII* mutant plaques was adapted here for *lacZ* mutant plaques. Figure 1 shows a brief overview of the workflow used in this experiment. After the *lacZ* positive selection assay was performed, *lacZ* mutant plaques from the bone marrow of each animal were collected using a transfer pipet and pooled together into microtubes containing autoclaved milliQ sterile water (0.3 mutants/μL; 1 sample per tube). All of the mutants from a single animal were pooled together in a single microtube. Thus, mutations were not assigned to individual plaques; rather, all mutations within an individual mouse were identified by alignment of each read against the *lacZ* gene. For a negative control, wild-type plaques with no expected mutations were collected and processed for sequencing. Microtubes containing pooled plaques were boiled for 5 min and then centrifuged at 18,000 g for 5 min. The supernatant was immediately transferred to a new microtube for storage, and 10 μL of the supernatant was transferred to another microtube for PCR. To control for the introduction of PCR errors, each pool of mutants was amplified in two separate reactions (i.e., two technical replicates per DNA pool). As the two replicates contain the same mutant DNA, it is expected that the same mutation should be present in both technical replicate sequences. The 30 μL PCR mastermix contained a final concentration of 1X PCR buffer, 1X Q solution, 200 nM of each primer, 50 μM dNTP, and 2.5 Units Taq DNA polymerase (Qiagen, Valencia, CA). The PCR primers used for amplification were custom designed using flanking DNA outside of the *lacZ* gene [GenBank:J01636.1]. The forward and reverse primers were GGCTTTACACTTTATGCTTC and ACATAATGGATTTCCTTACG, respectively. Amplification of the *lacZ* gene was carried out using the following thermocycle program: 95 °C for 3 min; 30 cycles of 95 °C for 45 s, 50 °C for 1 min, 72 °C for 4 min; final extension at 72 °C for 7 min. PCR products were purified using the QIAquick PCR purification kit (Qiagen) prior to library preparation.

### Ion Proton™ sequencing

Next-generation sequencing of the *lacZ* mutant products was done in-house using an Ion Proton™ sequencer (Life Technologies, Carlsbad, CA). The Ion Xpress™ Plus Library Kits and AB Library Builder™ (Ion Xpress Plus Library Protocol v. 1.00) were used to prepare one library for each animal’s pool of mutants from purified PCR products (for a total of 24 libraries). The procedure outlined in the user’s manual was followed with the exception of a 10 min custom shearing time and the replacement of Agencourt® AMPure® XP Reagent with SPRIselect® Reagent (Beckman Coulter, Brea, CA). The P1 Adapter and barcoded A adapters provided in the Ion Xpress™ Barcode Adapter kits were ligated to the ends of the fragmented libraries (i.e., each PCR product received a unique barcode). Gel electrophoresis of the libraries was performed on E-Gel® SizeSelect™ 2 % Agarose Gel using the E-Gel® iBase™ and visualized using the E-Gel® Safe Imager™ transilluminator. Fragments that were between ~175 and ~225 bp were excised from the gel and purified using the Invitrogen™ PureLink® Quick Gel Extraction Kit. Libraries were then amplified using the Library Amplification Primer Mix (Ion Plus Fragment Library Adapters kit) and Platinum® PCR SuperMix High Fidelity. Each amplified library was quantified/qualified using the Agilent® High Sensitivity DNA Kit and Agilent® Bioanalyzer® 2100 Instrument. Aliquots of each library were pooled together for a total final concentration of 16 pM. Emulsion PCR (em-PCR) of the pooled libraries was carried out using Ion Sphere™ particles on the Ion OneTouch™ 2 system. The Ion OneTouch™ ES was used to enrich for particles containing em-PCR products and the Qubit® 2.0 Fluorometer was used to assess the quality of enrichment. Enriched particles were deposited into the wells of an Ion P1™ chip (version 1) and sequenced by semi-conductor sequencing [21].

### Bioinformatics

The Proton™ Torrent Server version 3.6.2 interpreted the sequencing data and generated FASTQ files for each barcoded library. Reads were trimmed to remove low quality base calls and aligned to the reference sequence [GenBank: J01636.1] using Bowtie2 [22] (Settings: “– local” mode with “– very-sensitive-local”). The *lacZ* gene in our Muta™Mouse colony has four variations [6] relative to the reference coding sequence [GenBank:V00296.1] [23] that were corrected for.

Following alignment, a pileup format was prepared for each sample using SAMtools [24]. From the sample pileups, the number of reads that were wild-type or polymorphic (base substitutions/indels) was determined for each nucleotide position of the *lacZ* gene. At each nucleotide position, a proportion of reads is expected to carry a technical artefact (e.g., PCR artefacts and sequencing noise) for each possible mutation type (referred to as the false mutation proportion: see glossary in Table 1). However, these false mutation calls are consistent across samples (Additional file 1: Figure S1) and thus the false mutation proportion of each mutation type can be estimated individually at each nucleotide position. In order to prevent outlier libraries from influencing the background reading, false mutation proportions were estimated by calculating the median rate of occurrence of each mutation type across all samples for each nucleotide position. The data were then normalized by subtracting the false mutation proportion from the total mutation proportion to determine the true mutation proportion at each nucleotide position (Additional file 1: Figure S2; see glossary for clarification). Due to homopolymer sequencing errors, the false mutation proportion for some insertions/deletions (indels) can be much higher than false mutation proportions for base substitutions. To minimize false indel calls, indels appearing with a false mutation proportion higher than the highest false mutation proportion for base substitution calls were ignored.

**Table 1:** Glossary

Mutant – A plaque, grown on P-Gal media, that contains a loss of function mutation in the *lacZ* transgene.
Mutation – The change in DNA sequence (point mutation, indel) that knocks out the function of the *lacZ* transgene. There may also be silent mutations that can co-occur on the same *lacZ* copy as a loss of function mutation (“hitchhiker mutations”).
Clonal Expansion – When a cell containing a mutation produces daughter cells containing that same mutation, propagating it throughout the tissue.
False Mutation Proportion – At each nucleotide position, a proportion of the reads may contain a technical artefact and this proportion is consistent across samples. For example, if at position 25, the median frequency of G **→** A substitutions across all samples was one in every thousand reads, the false mutation proportion would be 0.001.
Total Mutation Proportion – At each nucleotide position, this is the mutation proportion for each mutation type within a single sample. For example, if sample 3 had a G **→** A substitutions at position 25 in 0.5 % of reads, the total mutation proportion would be 0.005.
True Mutation Proportion – The true mutation proportion of each mutation type at each position is determined by subtracting the false mutation proportion from the total mutation proportion. In the above example the true mutation proportion would be 0.004. This value is used to determine mutation counts.
Independent Mutations – Mutations that appear multiple times in a same sample due to clonal expansion are only counted as one mutation per sample. For example, if sample 1 had five G **→** A substitutions at position 25, this would be called a single independent mutation at position 25.
Recurrent Mutations – If a mutation occurs more than once within a sample it is considered recurrent as is likely the result of clonal expansion. In the previous example there are five G **→** A recurrent mutations at position 25.

Variants that remained following the false mutation proportion subtraction were considered mutations if the true mutation proportion was above threshold values determined based on the number of mutant plaques recovered from each sample and used in the sequencing (Additional file 1: Table S1). For example, if a mutant pool for an individual animal consisted of 100 plaques, a single mutation should be present in approximately 1 % of the sequenced reads (1 % of reads would have the variant while 99 % would have the reference nucleotide). To determine the threshold that should be set for identifying true mutations, the data were first analyzed using three different thresholds to optimize the sensitivity of the assay. The thresholds were set at: 1) the exact proportion for the mutation expected; 2) 75 % of the expected proportion; and 3) 50 % of the expected proportion. Using the previous example, the thresholds would be 0.01, 0.0075, and 0.005, respectively. In addition, in order for a mutation to be counted it needed to be present at values above the threshold in both technical replicates.

The average between the true mutation proportions of each technical replicate was used to provide a rough estimation of the total number of each type of mutation. For example, a base substitution present at a frequency of 0.10 would be counted as ten mutations in a sample that had a minimum threshold of 0.01. These values were used to determine the distribution of mutations across the *lacZ* gene for both the BaP-treated and control groups and identify mutation hotspots. In contrast, only independent mutations were used to determine the *lacZ* mutation spectra for both groups.

Finally, we examined the sequence sites of each specific mutation identified in the *lacZ* gene. The DNA codon table was used to determine whether base substitution changes observed in the coding sequence would result in amino acid changes in the protein. Based on this analysis, mutations were classified as nonsense, missense or silent.

### Statistical analysis

Statistical analyses were performed using R programming language to compare mutant frequencies and mutation spectra between control and treated animals [25]. The *lacZ* mutant frequencies in the bone marrow of control and BaP-treated animals were compared by Poisson Regression. Statistical comparisons of mutation types between control and treated groups were done using Pearson’s chi-squared test of independence.

**Fig. 2:**
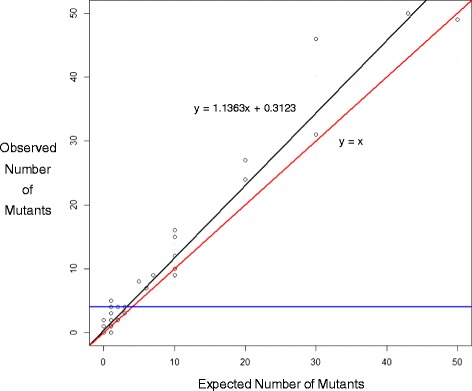
Correlation between observed and expected mutant counts. The *red line* shows a 1:1 ratio and the *black line* shows the true relationship between expected and observed mutant count. The plot shows that mutants with low counts cannot be accurately quantified. This results in a non-linear relationship between expected and observed mutant count (*P* < 0.0001). Hence, a limit of detection/linear model was used to adjust the total mutation counts of each independent mutation

Mutations that occurred more than once in the same animal were considered to be derived from clonal expansion (see glossary) from a single ‘independent’ event. The formula of [(total mutations – independent mutations)/total mutations] was used to estimate clonality. To test the method’s ability to estimate clonality, eight mutant plaques from different animals (each mutation identified by Sanger sequencing) were subcloned and then mixed together in different proportions (Additional file 1: Table S2). One of these mixtures contained different sized plaques to see whether plaque size affected clonality estimation. As an additional control, 100 random mutants from 100 different animals were pooled together to estimate the ability to identify unique mutations that were not expected to be clonally amplified. The observed proportions recovered by sequencing were then compared with the expected proportions of each mutant mixture. A Fitting Linear Models algorithm [26, 27] in R was used to estimate the limit of detection (LOD) for accurately quantifying mutant count using NGS. The algorithm searches for the LOD with the lowest squared error using 1000 iterations with decreasing step size. The model estimated that the LOD was four mutants (horizontal blue line in Fig. 2) and thus mutants with an observed count of four or less were set to a count of one. Above the LOD, the relationship between expected and observed mutant count is linear (*P* = 0.4249). Thus, using the LOD/Linear model we adjusted the count of each mutant in the BaP experiment for clonality measurements with the formula: Adjusted Mutant Count = (Observed Count – Intercept) / Slope for mutant counts > 4 where the y intercept = 0.3123 and the slope = 1.1363. Clonality was then calculated based on the adjusted mutant counts. For example, a count of three mutations was adjusted to one mutation and a count of 50 mutations was adjusted to 44 mutations.

## Results

### *lacZ* mutant frequency

As expected, bone marrow *lacZ* mutant frequencies were significantly higher in BaP-treated mice (701.7 ± 49.3 × 10^−5^) than control mice (8.6 ± 2.0 × 10^−5^) (P < 0.0001). This increase in mutant frequency is consistent with previous results [28]. However, the spontaneous mutant frequency was higher than expected based on historical controls (example: [28]), mostly due to animal Control20, which had a mutant frequency of 17.8 × 10^−5^. This animal was included in the study to test the method’s ability to detect clonal expansion.

### Sequencing output and read coverage

Sequencing of the pooled libraries using one Ion P1™ chip (version 1) gave 87,666,405 reads of 95 bp mean length for a total of 8.3 Gb. The number of usable reads was reduced to 66 % after removal of polyclonal Ion Sphere™ particles (29 %) obtained from the pooled library. Thus, the average nucleotide coverage per library was 9.1 ± 0.4 × 10^4^. The starts and ends of the reads across the gene appeared to be uniform (Additional file 1: Figure S3).

### False mutation proportion subtraction and threshold comparisons

An analysis of our data using conventional approaches revealed that most existing algorithms or variant callers were not equipped to detect rare mutations in pooled samples. Thus, we explored the use of a false mutation proportion subtraction methodology that we developed in-house. False mutation proportion refers to the sequencing artefacts that occur by chance at each nucleotide position. We use the term ‘proportion’ here to differentiate the proportion of reads carrying errors (background noise) from reads with true mutations. The false mutation proportions (see glossary for specific definition) for base substitutions ranged from 1.70 × 10^−5^ to 2.15 × 10^−2^ (median: 4.25 × 10^−3^), and from 1.64 × 10^−5^ to 4.64 × 10^−1^ (median: 7.52 × 10^−3^) for indels (Additional file 1: Figure S4). As the number of indel technical errors increase, the ability to reliably call mutations diminishes. Thus, indels were only called if the false mutation proportion was less than 2.15 × 10^−2^. Indels occurring at sites with a false mutation proportion greater than this were not included in the analysis. In the final analysis, this resulted in the removal of 21 out of 65 insertions (3/6 control, 18/59 BaP) and 99 out of 297 deletions (31/53 control, 68/244 BaP).

After correcting for the false mutation proportion, we explored mutation calling using three different thresholds for optimization (Additional file 1: Table S1). With the stringent threshold (i.e., the exact proportion expected), 152 and 854 independent mutations were recovered in both technical replicates for the control and BaP groups respectively (Table 2). The total number of mutations (unadjusted by LOD/Linear model) recovered out of 872 and 3000 mutant plaques from control and BaP-treated samples were 583 (67 %) and 2077 (69 %), respectively. Applying the two less stringent thresholds increased the number of mutations called from 2660 to 2912 and 3282, respectively; however, the discrepancies between technical replicates greatly increased. Thus, all downstream analyses were done on mutations called using the most stringent threshold. As expected, applying the most stringent threshold resulted in no mutation calls for the wild-type plaques.

**Table 2:** Summary of all mutations called using three different thresholds

		Independent Mutations			Total Mutations			^a^Mutants Removed		
	Threshold	^b^Base Subs.	^c^Ins	^d^Dels	Base Subs.	Ins	Dels	Base Subs.	Ins	Dels
Control	Stringent	127	3	22	542	7	34	14	101	117
	Medium	159	7	27	574	7	39	18	193	223
	Low	190	12	36	605	16	48	55	426	420
BaP	Stringent	637	41	176	1583	66	428	108	750	577
	Medium	767	60	239	1714	85	493	183	1139	818
	Low	921	125	338	1869	151	593	374	1846	1284

^a^Mutants removed refer to the number of variants that passed the threshold in one technical replicate but not the other

^b^Base substitutions

^c^Insertions

^d^Deletions

### Detection of clonal expansion

We created five control libraries (Additional file 1: Table S2) in which the number of mutations was known in order to determine the ability of our NGS approach to accurately predict the number of copies of each mutation. In addition, a library of 100 random mutants was sequenced to determine our ability to detect unique mutations. For each mutation detected, we divided the true mutation proportion by the expected mutation proportion for a single mutation (threshold) to estimate the number of copies of that mutation in any given sample. In the library of 100 random mutants there were 49 independent mutants detected with a total count of 102 recurrent mutations, which is a higher proportion of recurrent mutations than was expected [5]. It is possible that some of the recurrent mutations occurred at mutation hotspots. For example, five independent mutants and 15 recurrent mutations were detected at four of five known mutation hotspots [8]. However, it is more likely that some mutants were not detected while the count of the detected mutants was overestimated. When the expected mutant count was compared with the observed mutant count in the five control libraries (Fig. 2), we found that the count of each mutant was overestimated. Therefore, a limit of detection/linear model was developed to adjust the recurrent mutation counts of each independent mutation. Based on the model, the total mutation count for the library containing 100 random plaques was estimated to be 55 mutations. The mutant count estimation for the experimental libraries was 1652 mutant sequences (411 control, 1241 BaP) from 1006 independent mutations. A total of 917 mutants in the experimental libraries fell below the LOD, indicating a relatively small proportion of mutants underwent clonal expansion. The adjusted average clonality in the control and BaP samples was 43 and 31 % respectively (Additional file 1: Table S3), which is comparable to what was measured at the *cII* locus in the same mice (O’Brien, unpublished) and in other studies [2]. After adjusting for clonality, the *lacZ* mutation frequencies were 4.0 ± 1.0 × 10^−5^ and 484.0 ± 34.9 × 10^−5^ for control and BaP mice, respectively. The clonality adjustment revealed that the raw control mutant frequency was elevated in part by the presence of one outlier animal with extensive clonal expansion (86 %). After correcting for clonal expansion, the mutation frequency of this animal was in line with the mutation frequency of the rest of the animals (within one standard deviation of the mean; Additional file 1: Table S3). The model for adjusting clonality showed that BaP induction was 121-fold higher than control.

### *lacZ* mutation spectra and distribution

**Fig. 3:**
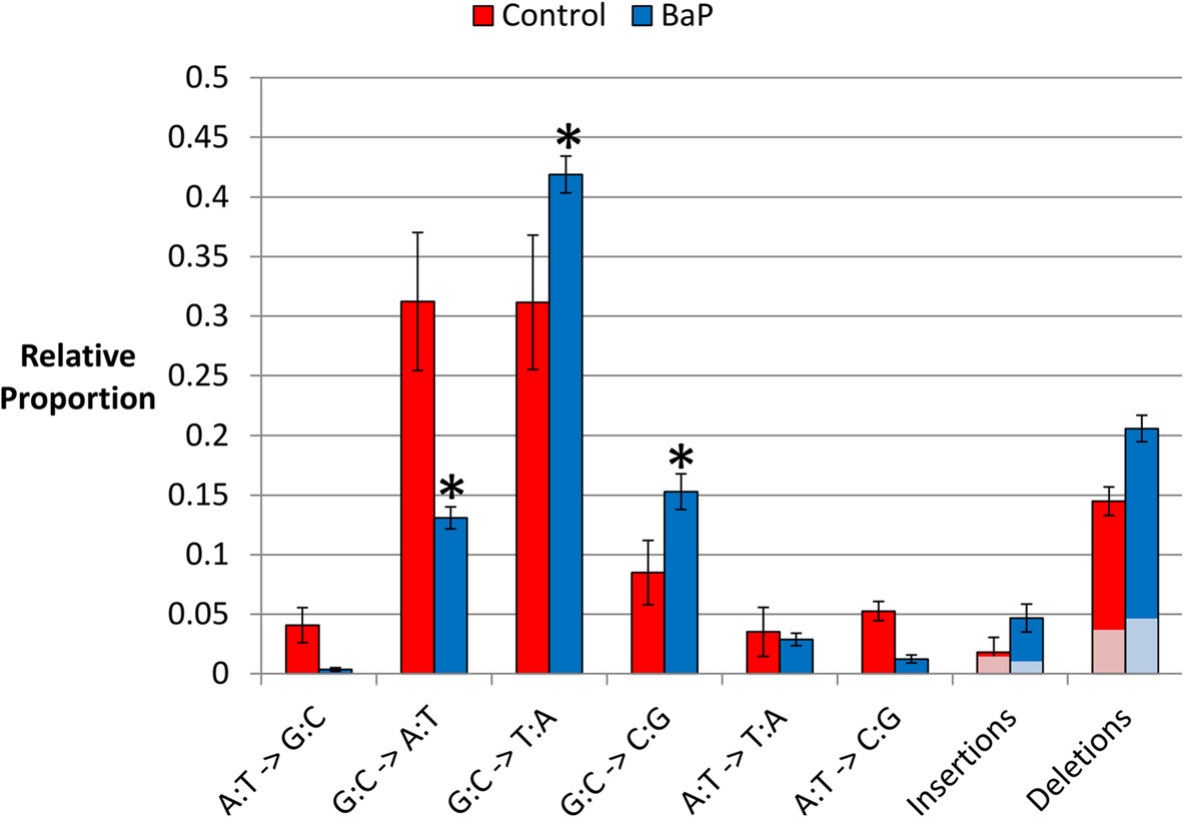
Relative proportion of control and BaP-induced *lacZ* mutations in bone marrow. Only independent mutations were considered. Mutation types with asterisks are significantly different between control and treated groups. The light-colored boxes within indels represent the fraction of indels that was inside or next to homopolymer stretches ≥ 3 bp long. The error bars represent the standard error between samples

The BaP mutation spectrum in bone marrow had significant differences from the control spectrum (Fig. 3). The proportions of transitions, transversions, and indels were 0.35, 0.49, and 0.16 in the control and 0.14, 0.61, and 0.25 in the BaP animals, respectively. The proportion of G:C **→** T:A and G:C **→** C:G transversions was significantly increased in BaP-treated animals relative to controls (*P* < 0.05). In addition, the proportion of G:C **→** A:T transitions was reduced (*P* < 0.0001) following BaP treatment. These findings are consistent with the *lacI* mutation spectrum in BaP-treated mouse liver [29] (Additional file 1: Figure S5). There were no significant differences in the proportion of the other mutation types between control and BaP-treated groups. When comparing the absolute frequencies of each mutation type, BaP exposure increased all mutation types above control except for A:T **→** G:C transitions (Additional file 1: Figure S6). To further test the accuracy of our NGS data, we compared the control and BaP-induced bone marrow *lacZ* mutation spectrum with published mutation spectra for transgene reporters from control and BaP-treated mice. The comparisons of control (Additional file 1: Figure S7) and BaP-induced (Additional file 1: Figure S8) spectra were consistent with the published data using Sanger sequencing, demonstrating that our NGS approach accurately identifies mutation spectra.

**Fig. 4:**
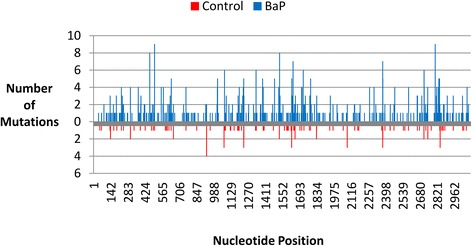
Distribution of unique base substitutions across the *lacZ* transgene for control and BaP samples. BaP base substitutions are on the positive y-axis and control substitutions are on the negative y-axis

Plotting the locations of the independent (Fig. 4) and recurrent (Additional file 1: Figure S9) base substitutions along the *lacZ* transgene to identify mutation hotspots demonstrated that the majority of induced and spontaneous mutations were at G:C base pairs (Additional file 1: Table S4). Within the *lacZ* transgene there are 12 short CpG sequences ≥ 5 nucleotides long. At these CpG sites there were five control point mutations (1G:C **→** A:T, 3G:C **→** T:A, 1G:C **→** C:G) and 36 BaP point mutations (1G:C **→** A:T, 24G:C **→** T:A, 11G:C **→** C:G). All nucleotide positions that had more than two independent mutations were at G:C sites in both control and BaP groups. Previously identified mutation hotspots in the *lacZ* gene using Sanger sequencing, based on ~500 mutations [5–10], were also hotspots in our dataset (Additional file 1: Figure S10). In all previous studies, there were 67 nucleotide positions with more than one mutation, and 51 (76 %) of these positions had a mutation in our dataset. We found mutations at all of the nucleotide positions that had more than four mutations across the previously published mutation hotspots. In the NGS data, there were 29 hotspots with more than four independent mutations across the different animals; of these nucleotide positions only two (7 %) were not identified using Sanger sequencing in previous studies.

**Fig. 5:**
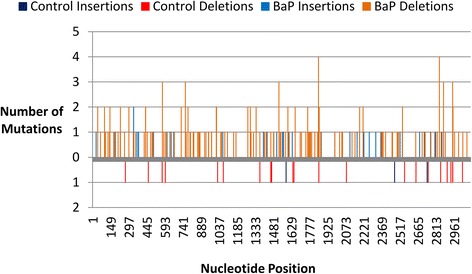
Distribution of unique indels across the *lacZ* transgene for control and BaP samples. BaP indels are on the positive y-axis and control indels are on the negative y-axis

The majority of indels that we observed were gains/losses of single nucleotides. There were 34 indels (of 242 in total) that involved two nucleotides: 32/198 deletions (3/22 control, 29/176 BaP); and 2/44 insertions (0/3 control, 2/41 BaP). Detecting indels was not as reliable as detecting base substitutions due to difficulty in sequencing homopolymer regions; sequencing accuracy of homopolymers falls to 97 % at homopolymer stretches of five or more base pairs [21]. In the *lacZ* gene, there are 591 short homopolymers (462 two mononucleotide repeats; 97 three mononucleotide repeats; and 32 ≥4 mononucleotide repeats). This means that 1353 bp (44 %) of the *lacZ* gene are within a short homopolymer. To test for a homopolymer bias, indels were plotted across the *lacZ* gene (Fig. 5, Additional file 1: Figure S11). The numbers of mutations occurring inside or adjacent to homopolymer stretches ≥ 3 bp long were: 2 out of 3 control insertions, 5 out 22 control deletions, 7 out of 41 BaP insertions, and 33 out of 176 BaP deletions (Fig. 3). Furthermore, only ten (31 %) of the 32 longest homopolymers in the *lacZ* gene had mutations within or adjacent to the homopolymers. Thus, the false mutation proportion subtraction accounted for many sequencing artefacts occurring around homopolymer regions.

Using the coding sequence of *lacZ*, we identified the amino acid changes that would result from each mutation. This analysis revealed that the proportion of missense, nonsense, and silent mutations was 39, 59, and 2 % in the control, and 50, 44, and 7 % in the BaP group, respectively. The silent mutations that were identified by NGS are likely “hitchhiker” mutations that occurred in the same plaque as a phenotype-inducing mutation. Sanger sequencing of mutant plaques shows that the proportion of plaques with more than one mutation (synonymous or non-synonymous) can be as high as 11 %, and the proportion of plaques with mutations separated by hundreds of nucleotides is around 3 % [6]. Also, the proportion of silent mutations observed here is consistent with the proportion of silent mutations detected using Sanger sequencing (<6 %) [5, 6, 8–10, 29]. The number of missense, nonsense, and silent mutations that occurred more than once at a nucleotide position was 10, 7, and 0 (20, 9, and 0 %) for the control, and 55, 62, and 4 (18, 22, and 8 %) for the BaP, respectively. Taking both groups into account there were 66, 81, and 4 positions where there was more than one missense, nonsense, and silent mutation, respectively. The low number of recurring silent mutations is as expected and reinforces the reliability of the data.

We also performed simulations, using random mutant sampling, to determine the minimum number of mutants per samples that would be sufficient to achieve a consistent mutation spectrum in the BaP group. In this simulation, mutations were sampled from 375, 348, 364, 360, and 299 BaP mutants (150, 142, 161, 138, and 139 BaP mutations) in five of the six treated animals, respectively. One animal had to be excluded because some of the mutation types did not occur frequently enough for accurate simulations. There were no significant differences in the mutation spectra of the five remaining animals (*P* = 0.3). As shown in Additional file 1: Figure S12, the results of our simulations demonstrated that sequencing approximately 100 mutants from a mouse provides an 80 % power to obtain the true mutation spectrum for that individual (*P* < 0.05).

## Discussion

We developed a simple, cost-effective, and high-throughput method to characterize the mutation spectrum of a transgenic mutation reporter locus in rodents using targeted NGS. This method can be adapted for any mutation reporter model using any NGS platform. In addition, the data analysis is simple and non-subjective. Using our conservative approach, we identified 1006 independent mutations within the *lacZ* transgene allowing us to characterize the spontaneous and BaP-induced mutation spectra with unparalleled precision. As expected, BaP exposure induced primarily G:C **→** T:A transversions. Our approach also allowed us to identify 1652 total mutations (including recurrent mutations that occurred more than once per mouse), permitting clonal expansion correction and making the assay 50 % more sensitive in detecting increases in mutation frequencies. Furthermore, comparisons of our NGS results with previous results obtained using Sanger sequencing show that the mutation spectra (Fig. 3), mutation hotspots (Additional file 1: Figure S10), and amino acid changes were as expected, demonstrating the robustness of our approach.

Characterization of the mutation spectra is important for identifying which sites are targeted by chemicals to elucidate mutagenic mechanism (e.g., what types of adducts are formed, faulty DNA repair processes, etc.). We investigated the mutation signature of BaP because it is a model carcinogen and its metabolic activation is well understood [30, 31]. BaP is metabolized by cytochrome P450 enzymes and epoxide hydrolase to mutagenic metabolites including benzo[*a*]pyrene-7,8-dihydrodiol-9,10-epoxide (BPDE) [31–33]. BPDE forms DNA adducts with the 2-amino group (N2) of guanine, and to a lesser extent with N6 and N4 of adenine and cytosine, respectively [32–34]. BPDE also produces reactive oxygen species through the spontaneous rearrangement of the epoxides or redox cycling, which also react with DNA leading to oxidative DNA damage, such as 8-oxo-deoxyguanosine [32]. These BaP damaging events explain the high proportion of guanine base substitutions, specifically G **→** T and G **→** C, seen here in bone marrow. In total, 89 % of BaP-induced mutations occurred at G:C nucleotides and all of the BaP-associated mutation hotspots were at G:C nucleotides. However, the refined mutation spectra obtained from sequencing thousands of mutants demonstrated that absolute mutation frequencies of all mutation types were elevated relative to the control with the exception of A:T **→** G:C transitions. Thus, BaP is a potent mutagen capable of inducing transversions at any nucleotide and transitions at G:C nucleotides.

One of the main reasons for sequencing mutants is to account for clonal expansion [3]. This can cause a tissue sample to have a higher mutant frequency than the rest of the samples within a group and falsely over-represents independent mutation events. To correct for clonal expansion, a subset of the mutant plaques from the tissue sample is sequenced to identify recurrent mutations arising from one progenitor cell that may be inflating the mutant frequency. Recurrent mutations are only counted as one mutation and the mutant frequency is adjusted accordingly (to reflect the true mutation frequency). Plotting the mutation distribution of all animals revealed potential clonal expansion events. Using the LOD/Linear model the corrected clonality estimates were 43 and 31 % in the control and exposed groups respectively. Correcting for clonality revealed that the fold increase in mutation frequency following BaP treatment was ~120-fold. This suggests that although NGS may overestimate the clonality of a tissue sample, the overestimation is consistent across samples and therefore a relative clonality comparison between groups can be achieved.

It should also be noted that the number of mutants sequenced can influence the clonal expansion estimation. In this study we sequenced a large number of mutants unmatched by any other study, and for this reason we may be seeing higher levels of clonality. Simulations show that had only 25 mutations been sequenced per animal (more typical of past experiments using Sanger sequencing [35]), the clonality would have remained at 43 % in the control group, but would have been reduced to 9 % in treated samples. The differences in clonality between actual and simulated measurements highlight the need to use caution when comparing clonality results determined by sequencing large versus small numbers of mutants. The lower clonality observed in the treated group can be explained by the presence of thousands of BaP-induced mutations that are reducing the chance of randomly sampling spontaneous mutations that were clonally expanded during mouse development.

The need to account for clonality was clearly demonstrated by the presence of one outlier control animal with a high mutant frequency in our study (Additional file 1: Table S3). Because of this outlier, which had 86 % clonality, our control group had a mean mutant frequency that was higher than expected based on previous studies [2]. However, after correcting for clonal expansion there was less variability in mutation frequencies within each group. Furthermore, the adjusted spontaneous mutation frequency was consistent with historical values for bone marrow from our laboratory [28, 36]. Finally, correcting for clonal expansion demonstrated that the mutation induction caused by BaP was underestimated by 50 % in the standard non-sequenced based approach. Had a weaker mutagen been tested, the transgenic rodent assay may have given a false-negative result without adjustment of clonal expansion. These examples show the added value of sequencing to measure clonality when interpreting the results of the TGR assay and demonstrate that our method can be used to correct for clonal expansion events at the *lacZ* locus to improve the reliability of reporter gene data.

There are notable strengths in our approach. First, our method uses a simple false mutation proportion subtraction to minimize sequencing artefacts and alignment errors. It is known that different NGS platforms and alignment tools have biases that can introduce false positives [37–40]. For example, the accuracy of semi-conductor sequencing decreases with increased homopolymer length [21], as is seen with pyrosequencing [41, 42]. However, these biases are consistent between the samples at each nucleotide position (Additional file 1: Figure S1) and thus can be corrected (Additional file 1: Figure S2). After applying the correction, we were able to identify independent mutations along the *lacZ* gene and approximate the number of times each mutation occurred. Second, we used technical replicates (two PCR amplifications) for each sample as a strategy to minimize the number of false-positives. This was done to account for errors that occurred during PCR as a result of Taq replication errors or thermal DNA damage [43, 44]. Using technical replicates does not reduce the throughput of the sequencing, and only marginally increases the cost of the experiment. Although this approach is very conservative and reduces the number of mutations called (i.e., we identified 1652 mutants from 3872 plaques), it is currently needed to eliminate false-positives, especially for indels, and accurately call mutations (Table 2). Thus, we strongly recommend that technical replicates be used with this method to control for PCR-based errors, allowing for accurate characterization of mutation spectra. Given the significant increases in the number of mutants that can be sequenced relative to Sanger sequencing, our stringent criteria for calling mutations does not reduce the power of the approach; indeed, we have sequenced an unprecedented number of *lacZ* mutations within a single experiment. This method was designed to be modular; therefore, researchers can explore the use of other bioinformatics and statistical tools that best fit their data. Before setting up this protocol in a laboratory, it is recommended that experiments be conducted using positive and negative controls to optimize parameters as we have done here.

The number of mutants that should be pooled per barcode must be considered carefully in the experimental design. As the number of mutants increase, the threshold to call a mutation decreases and the chance of identifying an artefact as a mutation increases. In addition, having too many mutants pooled per barcode will decrease the detection sensitivity of the NGS method. Contrarily, having too few mutants will not provide an accurate mutation spectrum. In order to approximate the number of mutants that should be pooled per barcode, we performed multiple data simulations based on the available results. These simulations show that sampling around 100 mutants per animal is sufficient to achieve reliable mutation spectra with a power of 80 %, and therefore, pooling approximately 170 mutants per barcode (assuming a mutant recovery of ~60 %) should be more than enough for any sequencing application. If sequencing of more mutations per sample is desired, additional DNA barcodes can be used. Another consideration is that the short length of the reads hinders the detection of large indels or complex mutations (multiple mutations occurring across the same DNA molecule). However, read lengths for the different platforms continue to increase [45] and may eventually increase to a point where complex mutations can be detected with a standard library.

Our method was designed using DNA barcoding to allow for cost- and time-effective analysis of *lacZ* mutation spectra. We estimate that the cost to sequence 3872 mutants using Sanger sequencing would be 20-fold higher than using NGS in reagent costs alone. Furthermore, this sequencing can be done in a week using NGS, while it would take at least 25 times as long using Sanger sequencing. If more mutants were sequenced, whether they were from the same samples or different experiments, the difference in cost and time would be greater. As demonstrated, there is an added bonus of sequencing large numbers of mutants: 1) mutation spectra can be characterized with high precision; 2) novel mutational hotspots can be identified (important for understanding an agent’s mode of action); and 3) silent mutations present on a mutated transgene, which are normally undetected, can be characterized. Considering that each barcoded library was sequenced with an average coverage of 91,000x (182,000x coverage per sample), several more samples could have been sequenced, making this approach highly efficient and amenable to the simultaneous sequencing of mutant plaques from different tissues and/or chemical agents. Furthermore, the cost and time to do these experiments will decline further as the throughput of NGS increases.

## Conclusions

In summary, we developed a high-throughput method for targeted-sequencing of long mutant reporter genes, specifically the *lacZ* transgene. The mutation spectra determined using the NGS analytical process are highly concordant with results using Sanger sequencing, demonstrating the accuracy of the methodology. After the animal exposure and tissue sample collection is complete, a whole experiment, from the beginning of the transgenic rodent assay to the end of the data analysis, can be done in less than 3 weeks using our newly established data analysis. The benefit of this method is that it can sequence as many mutants as the TGR assay can produce. Thus, this method has the potential to sequence hundreds to thousands of mutants from different tissues, exposure groups, and doses simultaneously. Despite the apparently low recovery of mutants by NGS due to the reduced sensitivity of calling mutations from pooled DNA, the rapid characterization of large numbers of mutants provides a more accurate measure of mutation spectra and clonal expansion. Although the method is applied to sequencing *lacZ* mutants, this approach can be modified to identify mutations in any reporter gene used in genetic toxicity testing. With the higher throughput and reduced cost offered by NGS, mutations induced by drugs or chemicals can now be more routinely assessed. Understanding the mode of action of different mutagenic agents enables improved regulation of genotoxins and ultimately a reduction in harmful diseases such as cancer.

### Availability of supporting data

Supporting data are available on the NCBI Sequence Read Archive under the BioProject accession number PRJNA284951.

## Additional file

Additional file 1:
**Figure S1.** Comparison of mutant read proportions for substitutions at each nucleotide position across all samples. **Figure S2.** Example density graphs (smoothed histograms) for variant calls at two different nucleotide positions. **Figure S3.** Distribution of read starts and ends. **Figure S4.** Comparison of the density of false mutation proportions between base substitutions (red) and indels (blue). **Figure S5.** Relative proportion of control and BaP-induced lacI mutations in liver from reference [29]. **Figure S6.** Bone marrow mutation spectra of control and BaP treatments. **Figure S7.** Comparison of spontaneous mutation spectra between our NGS dataset and other published datasets [4, 5, 7, 9-11]. **Figure S8.** Comparison of BaP-induced mutation spectrum in bone marrow measured using NGS with the mean mutation spectrum measured from four tissues (forestomach, spleen, colon, glandular stomach) using Sanger sequencing [6]. **Figure S9.** Distribution of non-unique base substitutions across the lacZ transgene for control and BaP samples. **Figure S10.** Comparison of lacZ mutation hotspots identified using Sanger sequencing [5-10] with mutations identified using NGS at the same nucleotide positions in the present study. **Figure S11.** Distribution of non-unique indels across the lacZ transgene for control and BaP samples. **Figure S12.** Results of simulations that use random sampling of BaP mutants to approximate the number of mutants per sample required to achieve a consistent mutation spectrum. **Table S1.** Thresholds used to detect mutations from each sample library. **Table S2.** Comparison of expected and observed mutant count when the number of each mutant plaque was controlled for in each NGS library. **Table S3.** Percent Clonality and Adjusted Mutant Frequencies in Control and BaP-treated samples using the LOD/linear model to adjust mutant counts. **Table S4.** Comparison of BaP-induced and spontaneous mutation spectra. (DOCX 418 kb)

## Abbreviations

BaP: Benzo[a]pyrene
NGS: Next-generation sequencing
TGR: Transgenic rodent
