# Supplementary material for: Characterizing Benzo[a]pyrene-induced lacZ mutation spectrum in transgenic mice using next-generation sequencing
Source: BMC Genomics. 2015 Oct 19;16:812. doi: 10.1186/s12864-015-2004-4 (PMC4617527; doi:10.1186/s12864-015-2004-4)
Supplement: Additional file 1: — Figure S1. Comparison of mutant read proportions for substitutions at each nucleotide position across all samples. Figure S2. Example density graphs (smoothed histograms) for variant calls at two different nucleotide positions. Figure S3. Distribution of read starts and ends. Figure S4. Comparison of the density of false mutation proportions between base substitutions (red) and indels (blue). Figure S5. Relative proportion of control and BaP-induced lacI mutations in liver from reference [29]. Figure S6. Bone marrow mutation spectra of control and BaP treatments. Figure S7. Comparison of spontaneous mutation spectra between our NGS dataset and other published datasets [4, 5, 7, 9-11]. Figure S8. Comparison of BaP-induced mutation spectrum in bone marrow measured using NGS with the mean mutation spectrum measured from four tissues (forestomach, spleen, colon, glandular stomach) using Sanger sequencing [6]. Figure S9. Distribution of non-unique base substitutions across the lacZ transgene for control and BaP samples. Figure S10. Comparison of lacZ mutation hotspots identified using Sanger sequencing [5-10] with mutations identified using NGS at the same nucleotide positions in the present study. Figure S11. Distribution of non-unique indels across the lacZ transgene for control and BaP samples. Figure S12. Results of simulations that use random sampling of BaP mutants to approximate the number of mutants per sample required to achieve a consistent mutation spectrum. Table S1. Thresholds used to detect mutations from each sample library. Table S2. Comparison of expected and observed mutant count when the number of each mutant plaque was controlled for in each NGS library. Table S3. Percent Clonality and Adjusted Mutant Frequencies in Control and BaP-treated samples using the LOD/linear model to adjust mutant counts. Table S4. Comparison of BaP-induced and spontaneous mutation spectra. (DOCX 418 kb) [file 12864_2015_2004_MOESM1_ESM.docx]

**SUPPLEMENTARY FIGURES**


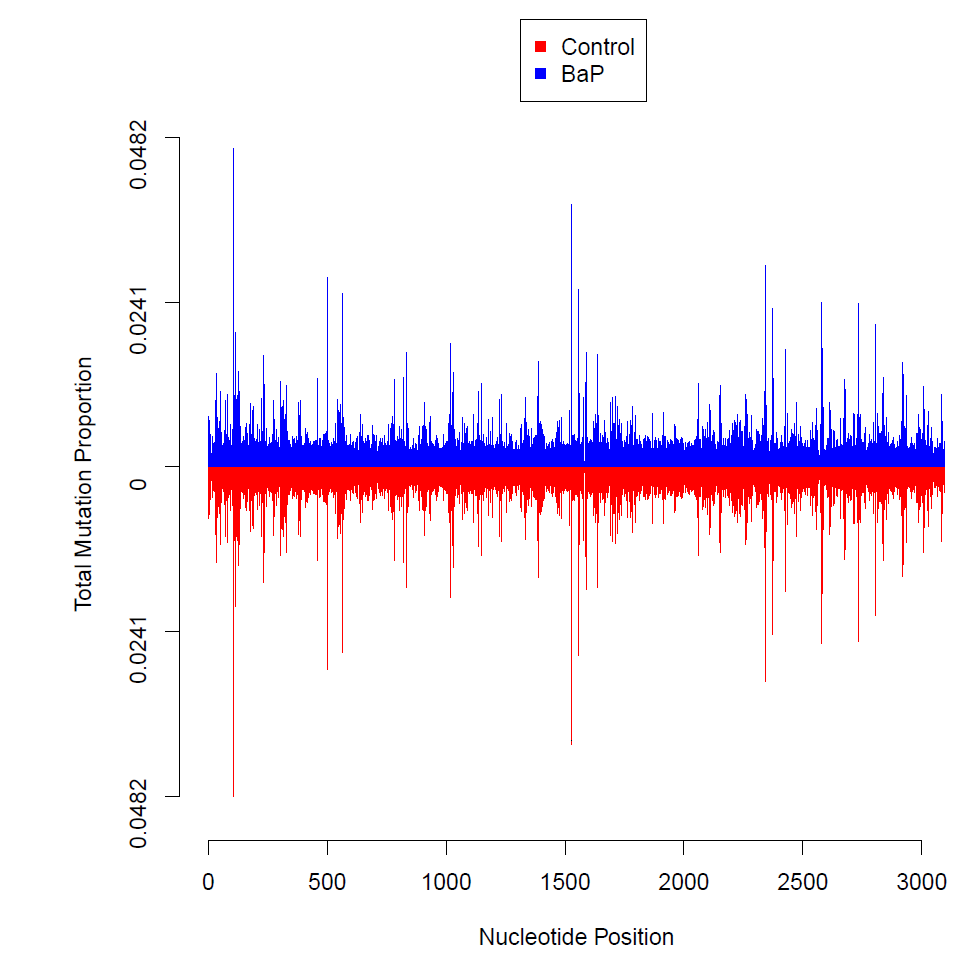


Supplementary Figure S1. Comparison of mutant read proportions for substitutions at each nucleotide position across all samples. This figure demonstrates that there is symmetry between total mutation proportions of control and treated animals for each nucleotide position. Thus, each nucleotide position had biases (false mutations) that were consistent between control and BaP samples. It is important to identify the false mutation proportion as we have done to discriminate between background sequencing noise and true mutations. Failure to do so would result in the same false positives (large peaks above) being called in every sample.


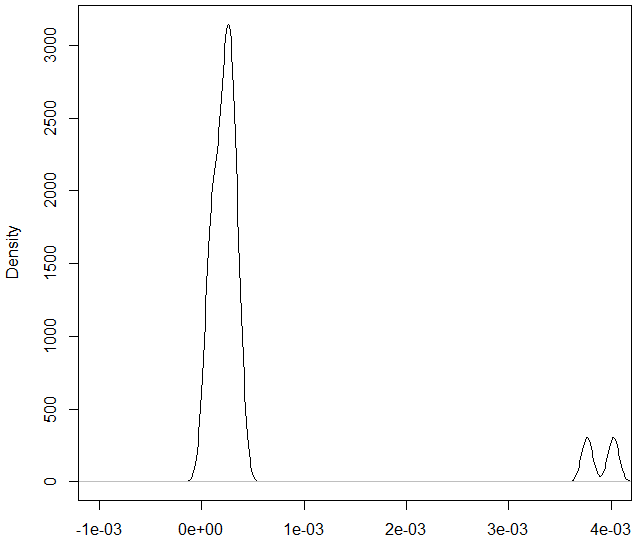

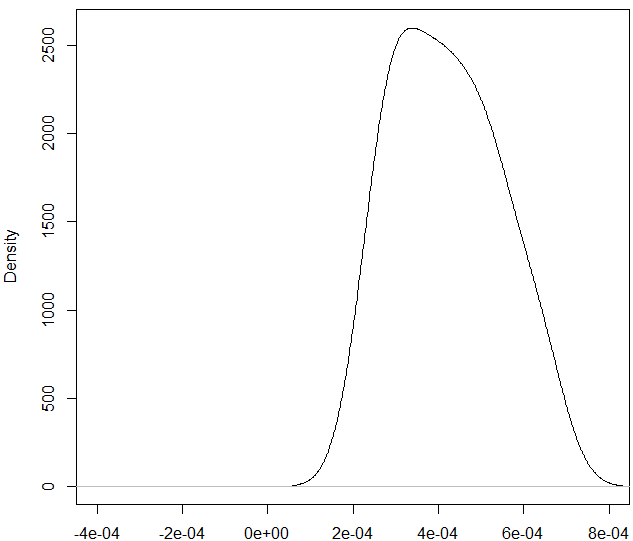
**A) Position 41: G** 🡪 **T B) Position 42: G** 🡪 **T**

Total Mutation Proportion

Total Mutation Proportion


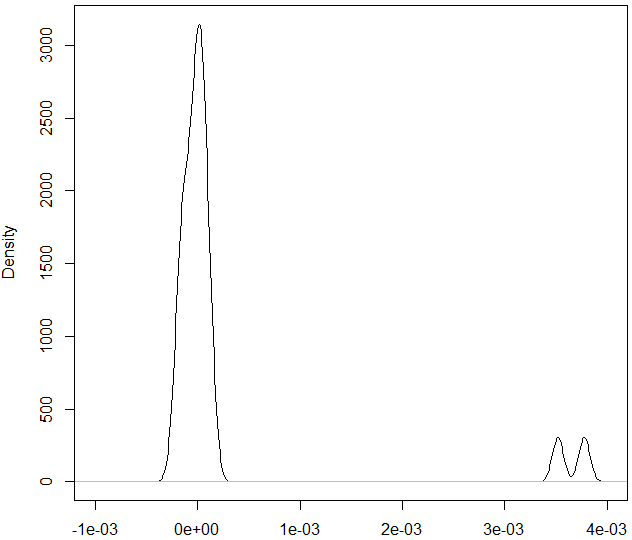

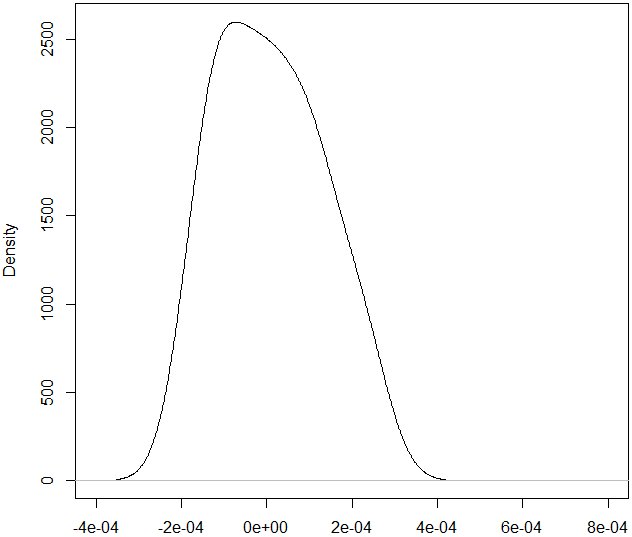


Total Mutation Proportion – False Mutation Proportion

Total Mutation Proportion – False Mutation Proportion

Supplementary Figure S2. Example density graphs (smoothed histograms) for variant calls at two different nucleotide positions. (A) Density graph when there is a true mutation (red arrow) present in one of the samples. In the top panel, the total proportion of reads carrying the G 🡪 T variant at position 41 across most samples is 2.4 x 10^-4^. This is the false mutation proportion (i.e., sequencing errors). By subtracting this false mutation proportion (bottom panel), the true mutation proportion (total mutation proportion – false mutation proportion) for most samples is now 0 (i.e., most samples have no mutations; shown by dotted blue line), except for the two peaks on the right of the graph. These peaks represent technical replicates of one sample and have true mutation proportions of 3.5 x 10^-3^ and 3.7 x 10^-3^. Considering that 500 mutant plaques were pooled together for this animal, a true mutation should be present in 1/500 or 2 x 10^-3^ reads. Since both technical replicates have true mutation proportions above this threshold value, the G 🡪 T variant was called as a true mutation. (B) Density graph when there is no mutation in any of the samples. The top panel shows that the total proportion of reads carrying the G 🡪 T variant across all samples is 4.1 x 10^-4^ for position 42. After applying the false mutation proportion subtraction, all samples cluster around 0 and there is no evidence of a mutation. Note, in the top panel the total mutation proportion for all samples was above 0 without correcting for background.


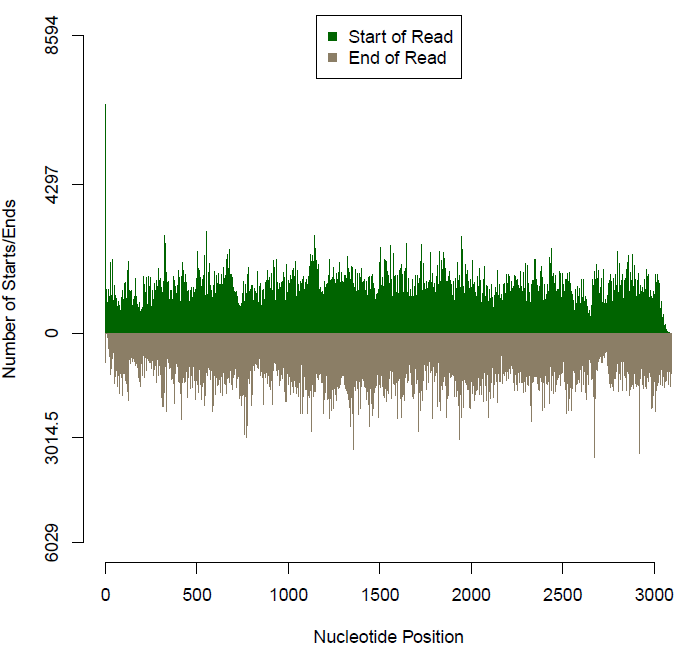


Supplementary Figure S3. Distribution of read starts and ends. The locations of read starts and ends appear to be uniform across the *lacZ* gene.


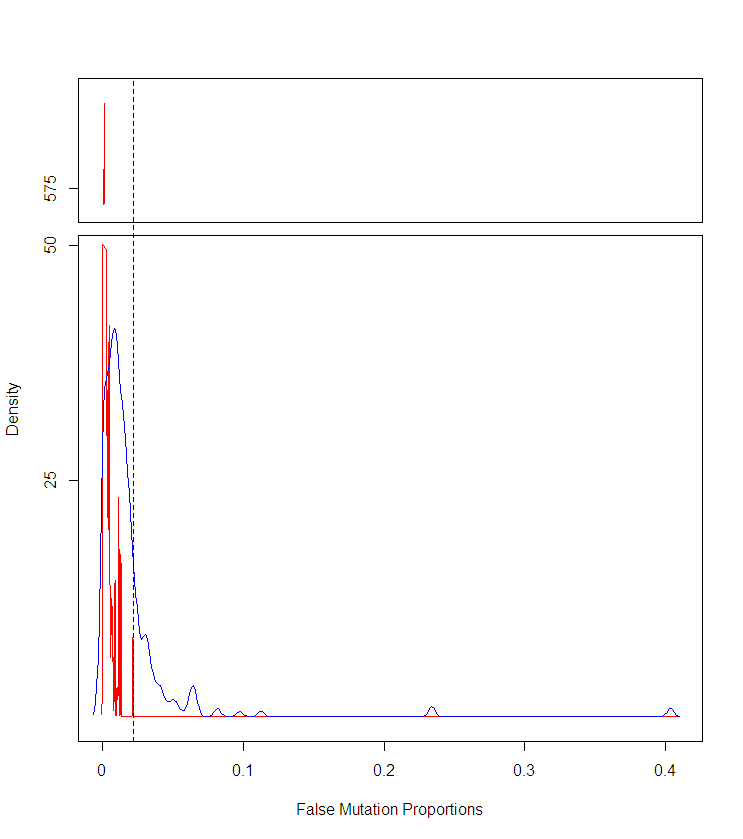

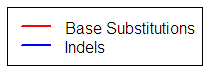


Supplementary Figure S4. Comparison of the density of false mutation proportions between base substitutions (red) and indels (blue). The highest false mutation proportion where a base substitution was called was 2.15 x 10^-2^ (dotted line). All indel calls at positions with false mutation proportions above 2.15 x 10^-2^ were at error-prone sequencing regions and were therefore ignored. Based on this graph, the indels with high false mutation frequencies were outliers and their removal was necessary.

Supplementary Figure S5. Relative proportion of control and BaP-induced *lacI* mutations in liver from reference [29]. The mutation spectra are based on 287 control mutants and 138 BaP mutants (66 from samples collected at hepatectomy, 72 from samples collected at sacrifice). Error bars for the BaP group represent the standard error between the two sets of BaP mutants.

Supplementary Figure S6. Bone marrow mutation spectra of control and BaP treatments. Only unique, independent mutations were considered and mutant frequencies were corrected for clonal expansion. All mutation types were significantly higher in the BaP samples (t-test: P<0.01) except for A:T 🡪 G:C transitions. The error bars represent the standard error between samples.

Supplementary Figure S7. Comparison of spontaneous mutation spectra between our NGS dataset and other published datasets [4, 5, 7, 9-11]. The spectrum reported from our NGS dataset is based on total mutation counts to be consistent with the previously published NGS dataset [11]. Insertions and deletions were combined and reported as “indels” in reference [11]; in order to include this information in the above graph, the proportion of indels was split evenly between insertions and deletions. The results between our NGS dataset and the other datasets are highly comparable, with G:C 🡪 A:T transitions being the most common mutation type. The results are based on 71 mutants from 3 tissues (liver, bone marrow, germ cells) [5], 24 male germ cell mutants [9], 89 bone marrow/liver mutants [10], 24 germ cell mutants [4], 321 mutants from six tissues (spleen, liver, heart, brain, skin, testis) [7], 591 lung mutants (Sanger) [11], 451 lung mutants (NGS)[11], and 572 bone marrow mutants (this study). Error bars are based on standard error between samples (this study), different mutant pools [11], or tissues (other studies).

Supplementary Figure S8. Comparison of BaP-induced mutation spectrum in bone marrow measured using NGS with the mean mutation spectrum measured from four tissues (forestomach, spleen, colon, glandular stomach) using Sanger sequencing [6]. Error bars are based on standard error between samples (this study) or tissues [6].

Supplementary Figure S9. Distribution of non-unique base substitutions across the *lacZ* transgene for control and BaP samples. BaP base substitutions are on the positive y-axis and control substitutions are on the negative y-axis. Nucleotide positions with several mutations are mostly due to clonal expansions.

Supplementary Figure S10. Comparison of *lacZ* mutation hotspots identified using Sanger sequencing [5-10] with mutations identified using NGS at the same nucleotide positions in the present study. All mutations in the graph correspond to base substitutions.

Supplementary Figure S11. Distribution of non-unique indels across the *lacZ* transgene for control and BaP samples. BaP indels are on the positive y-axis and control indels are on the negative y-axis. The 41 deletions at position 1527 are due to microsatellite deletions of CG at (CG)_4_ in three BaP samples.

Supplementary Figure S12. Results of simulations that use random sampling of BaP mutants to approximate the number of mutants per sample required to achieve a consistent mutation spectrum. The following steps were used: 1) different numbers of mutant plaques were randomly sampled from the available mutants in each BaP-treated animal; 2) the mutation spectrum (transitions, transversions, indels) was then determined using independent mutations that were sampled; and 3) the spectrum generated was compared to the true BaP spectrum for each animal using Pearson Chi-squared test. The percentage of simulations that gave the same spectrum (P ≥ 0.05) out of 10,000 iterations is plotted on the y-axis, the number of mutants sampled per animal are on the x-axis. Error bars are based on standard error between samples. The saturation curves show that the spectrum consistency has a power of 0.8 at approximately 100 mutants per animal. The spectrum consistency is maximal around 200 mutants per sample.

**SUPPLEMENTARY TABLES**

Supplementary Table S1. Thresholds used to detect mutations from each sample library.

| Sample ID | # of Mutant Plaques Sequenced | Stringent Threshold | Medium Threshold | Low Threshold |
| --- | --- | --- | --- | --- |
| Control15 | 125 | ^a^0.0080 | ^b^0.0060 | ^c^0.0040 |
| Control19 | 185 | 0.0054 | 0.0041 | 0.0027 |
| Control20 | 261 | 0.0038 | 0.0029 | 0.0019 |
| Control22 | 93 | 0.0108 | 0.0081 | 0.0054 |
| Control23 | 128 | 0.0078 | 0.0059 | 0.0039 |
| Control24 | 80 | 0.0125 | 0.0094 | 0.0063 |
| BaP43 | 500 | 0.0020 | 0.0015 | 0.0010 |
| BaP44 | 500 | 0.0020 | 0.0015 | 0.0010 |
| BaP45 | 500 | 0.0020 | 0.0015 | 0.0010 |
| BaP46 | 500 | 0.0020 | 0.0015 | 0.0010 |
| BaP47 | 500 | 0.0020 | 0.0015 | 0.0010 |
| BaP48 | 500 | 0.0020 | 0.0015 | 0.0010 |

^a^ 1/125 = 0.008

^b^ 0.008 * 75% = 0.006

^c^ 0.008 * 50% = 0.004

Supplementary Table S2. Comparison of expected and observed mutant count when the number of each mutant plaque was controlled for in each NGS library.

|  | 2413: Deletion | | 2286:  G 🡪 A | | 1224:  C 🡪 A | | 2374^a^:  C 🡪 A | | 491:  G 🡪 A | | 572: Deletion | | 1546:  C 🡪 T | | Titre | Total Plaques |
| --- | --- | --- | --- | --- | --- | --- | --- | --- | --- | --- | --- | --- | --- | --- | --- | --- |
|  |  |  |  |  |  |  |  |  |  |  |  |  |  |  |  |  |
| Library | E | O | E | O | E | O | E | O | E | O | E | O | E | O |  |  |
| 1 | 10 | 12 | 10 | 9 | 10 | 10 | 20 | 24 | 10 | 12 | 10 | 15 | 10 | 16 | 0 | 80 |
| 2 | 1 | 1 | 1 | 2 | 2 | 2 | 5 | 8 | 3 | 3 | 3 | 4 | 30 | 46 | 52 | 97 |
| 3 | 5 | 8 | 6 | 7 | 7 | 9 | 43 | 50 | 20 | 27 | 30 | 31 | 50 | 49 | 27 | 188 |
| 4 | 1 | 2 | 1 | 2 | 1 | 2 | 2 | 4 | 1 | 0 | 1 | 1 | 1 | 3 | 90 | 98 |
| 5^b^ | 0 | 0 | 0 | 0 | 0 | 0 | 10 | 11 | 0 | 0 | 10 | 5 | 10 | 20 | 30 | 60 |

^a^The same mutation was observed twice in two separate mutants at the hotspot nucleotide position 2374.

^b^Different plaque sizes were sequenced for each mutant. Small, medium, and large mutants were sequenced from mutants at positions 572, 2374, and 1546 respectively.

Supplementary Table S3. Percent Clonality and Adjusted Mutant Frequencies in Control and BaP-treated samples using the LOD/linear model to adjust mutant counts.

| Sample | Raw Mutant Frequency (x 10^-5^) | Average Raw Mutant Frequency (x 10^-5^) | Clonal Expansion (%) | Adjusted Mutant Frequency (x 10^-5^) | Average Adjusted Mutant Frequency  (x 10^-5^) |
| --- | --- | --- | --- | --- | --- |
|  |  |  |  |  |  |
| Control15 | 5.8 | 8.6 | 61.8 | 2.2 | 4.0 |
| Control19 | 9.7 |  | 69.2 | 3.0 |  |
| Control20 | 17.8 |  | 85.5 | 2.6 |  |
| Control22 | 3.7 |  | 28.1 | 2.7 |  |
| Control23 | 6.2 |  | 14.8 | 5.3 |  |
| Control24 | 8.3 |  | 0 | 8.3 |  |
| BaP43 | 590.2 | 701.7 | 33.6 | 391.9 | 484.0 |
| BaP44 | 725.9 |  | 31.4 | 498.0 |  |
| BaP45 | 865.2 |  | 35.1 | 561.5 |  |
| BaP46 | 782.9 |  | 31.5 | 536.3 |  |
| BaP47 | 538.7 |  | 32.4 | 364.2 |  |
| BaP48 | 707.3 |  | 21.9 | 552.4 |  |

Supplementary Table S4. Comparison of BaP-induced and spontaneous mutation spectra.

| Mutated Nucleotide | Control | | | BaP | | |
| --- | --- | --- | --- | --- | --- | --- |
|  | ≥ 1 Independent Mutation^a^ | ≥ 2 Independent Mutations | >2 Independent Mutations^b^ | ≥ 1 Independent Mutation | ≥ 2 Independent Mutations | >2 Independent Mutations |
|  |  |  |  |  |  |  |
| G:C | 83% | 88% | 100% | 89% | 98% | 100% |
| A:T | 17% | 12% | 0% | 11% | 2% | 0% |

^a^The majority of mutations occurred at G:C base pairs.

^b^All the nucleotide positions that had more than 2 independent mutations were at G:C base pairs.
